# Elective lymph node irradiation late course accelerated hyper-fractionated radiotherapy plus concurrent cisplatin-based chemotherapy for esophageal squamous cell carcinoma: a phase II study

**DOI:** 10.1186/1748-717X-8-108

**Published:** 2013-05-02

**Authors:** Dongqing Wang, Jiali Yang, Jingyu Zhu, Baosheng Li, Limin Zhai, Mingping Sun, Heyi Gong, Tao Zhou, Yumei Wei, Wei Huang, Zhongtang Wang, Hongsheng Li, Zicheng Zhang

**Affiliations:** 1 Department of Radiation Oncology, Shandong Cancer Hospital, Shandong Academy of Medical Sciences, Jinan, China; 2 Department of Gastroenterology, Qidu Hospital affiliated to Qingdao Medical College, Zibo, China; 3 Jinan Central Hospital affiliated to Shandong University, Jinan, China

**Keywords:** Esophageal squamous cell carcinoma, Elective lymph node irradiation, Chemoradiotherapy

## Abstract

**Background:**

In this phase II study, we evaluated the efficacy, toxicity, and patterns of failure of elective lymph node irradiation (ENI) late course accelerated hyper-fractionated radiotherapy (LCAHRT) concurrently with cisplatin-based chemotherapy (CHT) for esophageal squamous cell carcinoma (ESCC).

**Methods:**

Patients with clinical stage II-IVa (T_1-4_N_0-1_M_0_ or M_1a_) ESCC were enrolled between 2004 and 2011. Radiation therapy (RT) comprised two courses: The first course of radiation covered the primary and metastatic regional tumors and high risk lymph nodal regions, given at 2 Gy per fraction for a dose of 40 Gy. In the second course, LCAHRT was delivered to the boost volume twice a day for an additional 19.6 Gy in 7 treatment days, using 1.4 Gy per fraction. Two cycles of CHT were given at the beginning of RT.

**Results:**

The median age and Karnofsky performance status were 63 years and 80, respectively. The American Joint Committee on Cancer stage was II in 14 (20.6%) patients, III in 32 (47.1%), and IV_a_ in 22 (32.3%). With a median follow-up of 18.5 months, the overall survival at 1-, 3-, 5-year were 75.5%, 46.5%, 22.7% for whole group patients, versus 78.6%, 49.4%, 39.9% for patients with stage II–III. The patterns of first failure from local recurrence, regional failure, and distant metastasis were seen in 20.6%, 17.6%, and 19.1%, respectively. The most frequent acute high-grade (≥ 3) toxicities were esophagitis and leucopenia, occurred in 26.4% and 32.4%.

**Conclusions:**

ENI LCAHRT concurrently with CHT was appeared to be an effective regimen for ESCC patient with a favorable and tolerated profile. Further observation with longer time and randomized phase III trial is currently underway.

**Trial registration:**

ChiCTR-TRC-09000568

## Background

Based on the results of the Radiation Therapy Oncology Group (RTOG) phase III intergroup trial 85–01 and 95–04, the standard therapy for patients with localized esophagus carcinoma selected for nonsurgical treatment is radiation therapy (RT) plus concurrent chemotherapy [1,2]. In RTOG 85–01, combined therapy significantly increased 5-year overall survival (OS) to 26% (95% confidence interval [CI], 15%–37%) compared with RT alone. However, the incidence of local/regional failure and local/regional persistence of disease was high up to 45.9% [1]. In an attempt to improve these results, RTOG 95–04 increased radiation dose from 50.0 Gy to 64.8 Gy, however, intensification of the radiation dose did not improve local/regional control or survival [2]. Although the reason for the lack of benefit using higher dose is unclear, a significant prolongation of treatment time may have contributed, in part, to this result [2].

Several animal experiments and clinical investigations have shown that accelerated proliferation of surviving tumor clonogen during a standard schedule of RT is one of the major reasons for treatment failure [3,4]. It is postulated theoretically that a shortened irradiation course, still keeping the total radiation dose, or increased dose of radiation delivered in the late course of the treatment would improve local control for esophageal carcinoma by overcoming the deleterious effects of accelerated repopulation. Clinical data from China [5] has shown late course accelerated hyperfractionated radiotherapy (LCAHRT) improved the 5-year OS (odds ratio [OR] = 2.93, 95% CI: 2.15–4.00, p < 0.00001) and 5-year local control (OR = 3.96, 95% CI: 2.91–5.38, p < 0.00001) than standard fractionated RT in the localized esophageal carcinoma. Clinical investigation in nasopharyngeal [6,7] and lung carcinoma [8] also displayed promising treatment outcome.

For esophageal squamous cell carcinoma (ESCC), lymph nodal failure remains a major reason for poor prognosis. Since the early 1980s, Japanese surgeons have practiced 3-field regional lymph node dissection for esophageal cancer and led to an improved survival [9,10]. It is thought that prophylactic 3-field lymph node dissection improves the survival rate by eliminating micrometastases and reducing the regional lymph node recurrence rate. In accordance with the concept of 3-field lymph node dissection in curative surgery, elective nodal irradiation (ENI) has been adopted for definitive chemoradiotherapy in Japan. Although, the benefit of ENI for ESCC remains controversial, recently studies from Japan [11,12] have confirmed ENI was effective for preventing regional and distant nodal failure in patients with esophageal carcinoma undergoing concurrent chemoradiation.

In randomized clinical trials, no consistent benefit was seen for any specific chemotherapy regimens for locally advanced ESCC. Cisplatin is one of the most active agents, with a single agent response rate consistently in the range of 20% or greater. Older agent 5-fluorouracil and newer agents such as capecitabine, pemetrexed, were reported effectiveness for locally advanced ESCC [13].

Based on above thinking, our institution had engaged in clinical trials of ENI LCAHRT concurrently with cisplatin-based chemotherapy (CHT) for ESCC since 2004. The results of our phase I studies [14,15] demonstrated that this treatment scheme was feasible and tolerated, and the treatment outcomes were encouraging. The results with longer follow-up are now reported in the present study.

## Methods

### Patients population

Sixty-eight ESCC patients receiving ENI LCAHRT concurrently with CHT were enrolled from January 2004 to November 2011. Enrollment was limited to clinical stage T_1_ to T_4_, N_0/1_, M_0/1a_ of primary ESCC located at cervical-, upper-, mid-, or distal-esophagus, according to American Joint Committee (AJCC) tumor-node-metastasis (TNM) system (2002). Eligibility and exclusion criteria were seen below. The protocol was approved by our institutional review board, and written informed consent was obtained from all patients.

### Eligibility criteria

The eligibility criteria were as following: (1) a Karnofsky Performance Status (KPS) ≥ 70; (2) patients ≤ 75 years old; (3) histologically confirmed ESCC with the previously untreated; (4) TNM stage for II, III, and IV_a_ (cervical or celiac node metastasis, not include organ metastasis according to AJCC 2002); (5) weight loss ≤ 5%; (6) life expectancy ≥ 3 months; (7) absolute white blood cell count ≥ 4000/ml, platelets ≥ 100,000/ml, total bilirubin level ≤ 1.5 mg/dl, serum creatinine level ≤1.5 times the upper limit of normal, and aspartate/alanine aminotransferase levels ≤ 2.5 times the upper limit of normal. The TNM stage was assessed by enhanced computed tomography (CT) and/or combined with ^18^F-fluorodeoxyglucose position emission tomography/computed tomography (^18^F-FDG PET/CT).

### Exclusion criteria

Exclusion criteria were distant organ metastases, radiographic or bronchoscopic evidence of esophageal peroration, the minimum lumen size less than 5 mm, and some other serious underlying medical conditions such as significant cardiac disease, uncontrolled diabetes, and previous evidence of chemotherapy or RT.

### Pretreatment evaluation

Pretreatment evaluation included a complete history and physical examination, assessment of KPS and quality of life, serum chemistry profile, complete blood cell count, chest x-ray, ECG, endoscopy with biopsy. In order to exclude patients with distant organ metastases, pretreatment evaluation also included upper gastrointestinal, chest, and abdominal CT scan, bone scan with single photon emission computed tomography, magnetic resonance imaging scan of the brain and neck, or a whole body ^18^F-FDG PET/CT scan.

### Chemoradiotherapy

Details of LCAHRT were published previously [14,15]. Briefly, the radiation was carried out by 6 MV or 15 MV X-ray using a two-course irradiation schedule: the first course of radiation covered the primary tumors and metastatic regional lymph node(s) and high risk lymph nodal regions (HRLNR), given at 2 Gy per fraction, 5 days a week for a dose of 40 Gy in 20 fractions; the second course of radiation was delivered to the boost volume for an additional dose of 19.6 Gy twice a day in 14 fractions within 7 days using 1.4 Gy per fraction with a 6 h minimal interval between fractions. The total dose delivered of the two-course irradiation would be 59.6 Gy/34 fractions in 5.4 weeks. Accelerated radiation (1.4 Gy per fraction) was used in this protocol in an effort to prevent the late toxicities of mormal tissues. The gross tumor volume included primary cancer (GTV_p_) and metastatic lymph nodes (GTV_n_). The planning target volume (PTV) of the first course (PTV_1_) was defined as GTV_p_ adding a 5 cm margin superiorly and inferiorly and 1 cm laterally, and GTV_n_ with a 0.8 cm margin, as well as HRLNR adding a 0.8 cm margin. The PTV of the second course (PTV_2_) was shortened along the superior and inferior ends of GTV_p_ with 3 cm margins, whereas the width of fields remained 1 cm, as well as GTV_n_ adding a 0.8 cm margin. However, the HRLNR were spared.

The chemotherapeutic regimens in present study consisted of cisplatin 25 mg/m^2^/day i.v. on days 1–3 plus 5-FU 600 mg/m^2^/24h by continuous infusion on days 1–5 or plus capecitabine 1000 mg twice daily with a 12 h interval on days1–14 or plus pemetrexed 400 mg/m^2^ on days 1 of a 21-day cycle [14,15]. Two cycles of concurrent chemotherapy were administrated during the process of RT. The choice of chemotherapy regimens for this study was dependent on the economic situation of patients and their voluntary.

### Treatment-related toxicity assessment and follow-up

Treatment-related toxicity assessment was performed at least weekly during treatment, 4 weeks after completion of therapy, every 3 months for 2 years, and every 6 months thereafter using the National Cancer Institute Common Toxicity Criteria (version 3.0). A full history and physical examination, as well as repeat blood work were recorded at these visits. A Spiral CT-scans of the neck, chest, and abdominal were obtained at every follow-up examination to monitor morphological changes in normal tissue structure with respect to radiation-induced toxicity and to evaluate the status of locoregional and distant disease.

### End points and statistics

The primary end point for this study was OS. Survival was measured from the beginning of RT until the last follow-up or death. The secondary end points were treatment-related toxicity, and patterns of failure, included local recurrence (primary tumor), regional failure (lymph nodes), locoregional failure (local recurrence plus regional failure) and distant metastasis. The survival analysis was performed by the actuarial Kaplan-Meier method, and differences between the curves were analyzed by using the log-rank test. Analysis of patterns of failure was performed by using crude calculations.

## Results

### Patient-, tumor- and treatment-related characteristics

**Figure 1:**
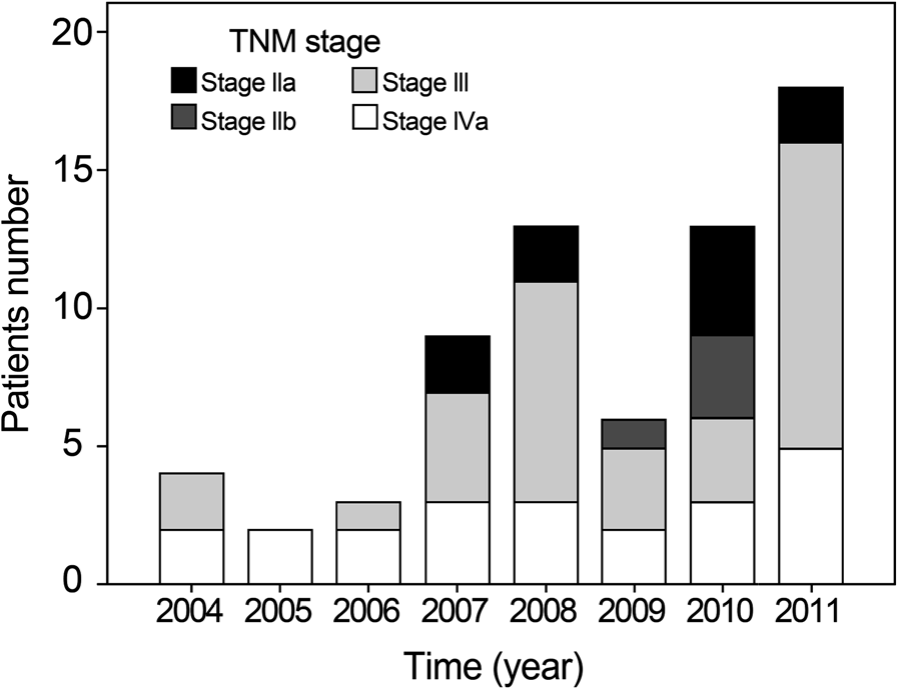
**TNM stage distribution in 68 patients.** The American Joint Committee TNM stage was II in 14 (20.6%) patients, III in 32 (47.1%), and IV_a_ in 22 (32.3%), respectively.

Sixty-eight patients (male/female 58/10; cervical/upper/mid/lower 8/24/27/9) were enrolled in the study. All patients were followed until death or the time of analysis. The age ranged from 40 to 75 years (median, 63 years). The KPS was evaluated as 70–100 (median, 80). Fourteen patients had stage II, 32 stage III, and 22 stage IV_a_. The distribution of TNM stage for enrolled patients from 2004 to 2011 was displayed in Figure 1. Patient-, tumor- and treatment-related characteristics were summarized in Table 1.

**Table 1:** Patient-, tumor- and treatment-related characteristics

**Characteristics**	**No.**	**%**
No. of patients	68	100
Age (years)		
Median	63	—
Range	40–75	—
Gender		
Male	58	85.3
Female	10	14.7
Performance status		
KPS 70-80	52	76.5
KPS 90-100	16	23.5
AJCC TNM stage		
Stage II_a_	10	14.7
Stage II_b_	4	5.9
Stage III	32	47.1
Stage IV_a_	22	32.3
Tumor length (cm)		
Median	5.0	—
Range	2.0–11.5	—
Tumor location		
Cervical	8	11.8
Upper-thoracic	24	35.3
Mid-thoracic	27	39.7
Lower-thoracic	9	13.2
*Regimens		
5-fluorouracil	20	29.4
Capecitabine	12	17.5
Pemetrexed	32	47.1

* Others treated with cisplatin plus vinorelbineis (1), gemcitabine (1), and docetaxel (2).

### Survival

**Figure 2:**
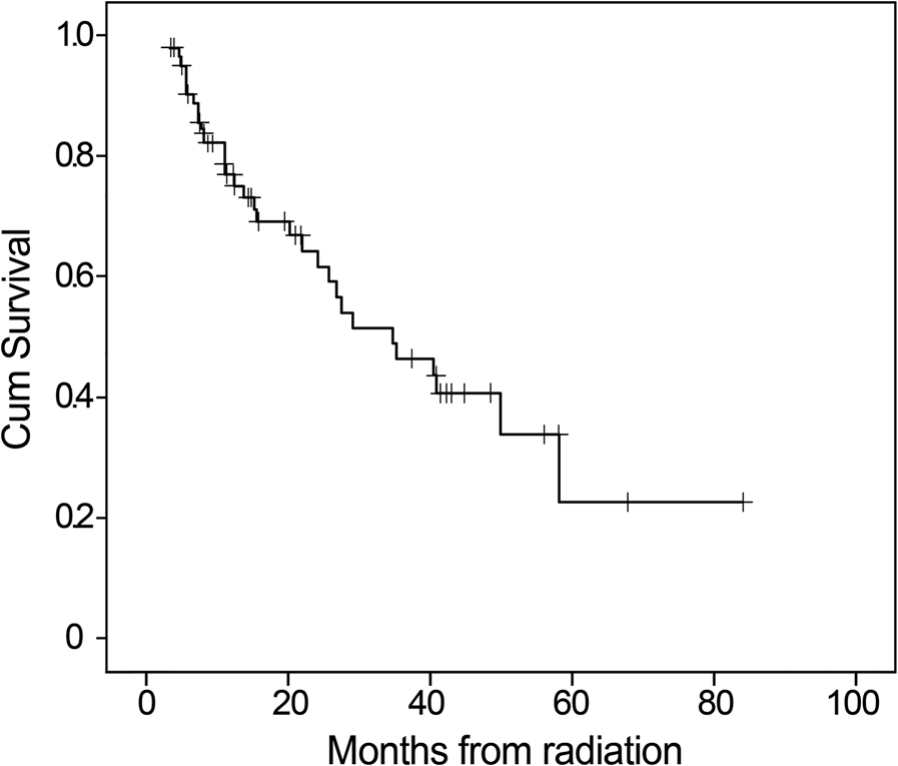
**Kaplan-Meier curve of overall survival (OS) for whole group patients treated with late course accelerated hyper-fractionated radiotherapy plus concurrent chemotherapy.** With a median follow-up of 18.5 months, the median survival was 34.4 months (95% confidence interval: 19.1–49.6 months), and the 1-, 3-, 5-year OS were 75.5%, 46.5%, 22.7%, respectively.

**Figure 3:**
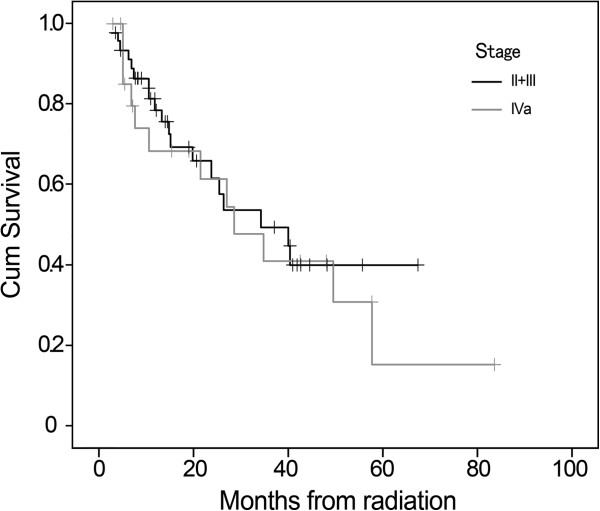
**Kaplan-Meier curves of overall survival (OS) were compared in patients with TNM stage II-III and IV**_**a**_**.** For patients with stage II–III, the 1-, 3-, and 5-year OS were 78.6%, 49.4%, and 39.9%, respectively, versus 68.3%, 41.0%, and 15.4% for IV_a_ patients, respectively, however, no statistically significant difference was observed in our limited sample size (p=0.671).

**Figure 4:**
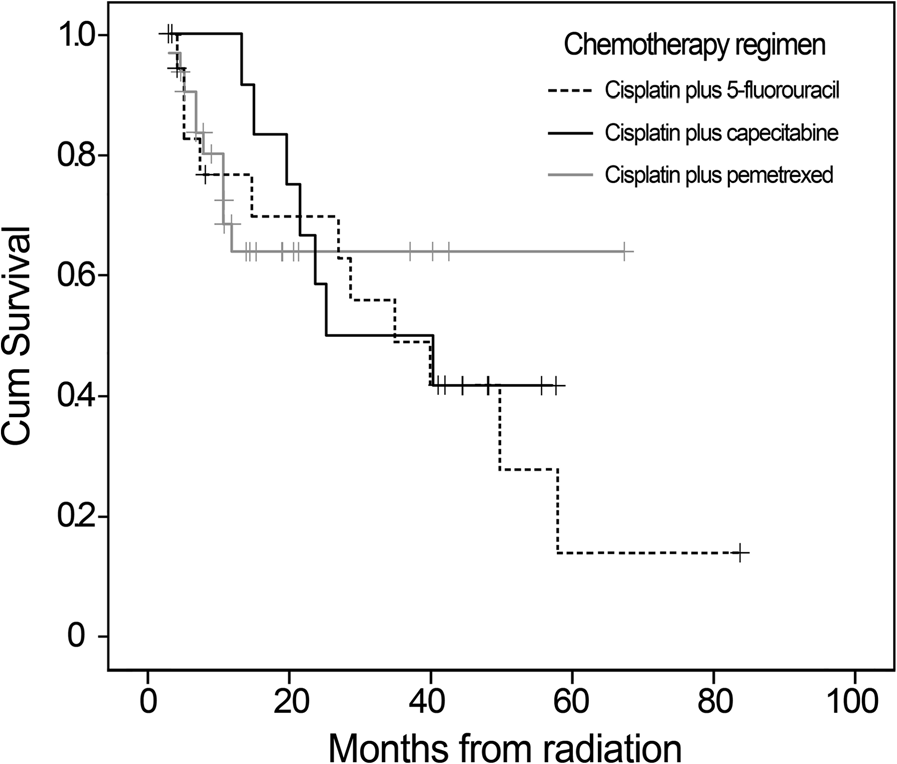
**Kaplan-Meier curves of overall survival (OS) were compared in patients receiving different chemotherapy regimens.** Our data did not show statistically significant difference in OS rates between the three groups (p=0.690).

The median survival was 34.4 months (95% CI 19.1–49.6 months) for whole group patients, and the 1-, 3-, 5-year OS were 75.5%, 46.5%, 22.7%, respectively (Figure 2). For patients with stage II–III, the 1-, 3-, and 5-year OS were 78.6%, 49.4%, and 39.9%, respectively, versus 68.3%, 41.0%, and 15.4% for IV_a_ patients, respectively. The Kaplan-Meier survival curve was presented in Figure 3. The difference in OS among patients with different clinical stages was not statistically significant (Chi-square=0.180, p=0.671). Similar result was also observed among patients receiving different chemotherapy regimens (Chi-square=0.159, p=0.690) (Figure 4).

### Patterns of failure

The crude patterns of failure were listed in Table 2. The incidence of any local recurrence, regional failure, and distant metastasis were 20.6%, 17.6%, and 19.1%, respectively. About twenty-nine percent (20/68) of the patients had local or/and regional disease presenting as the first failure, distant metastases as the first failure occurred in 13 patients. The sites of distant metastases were: liver (4), bone (2), lungs (1), pleura (1), brain (1), and multi-organs (1). Moreover, another three patients developed distant lymph node metastases containing cervical (1), celiac (1), and retroperitoneal (1) parts.

**Table 2:** Patterns of failure

**Patterns of failure**	**No.**	**%**
Local recurrence only	8	11.8
Regional failure only	6	8.8
Locoregional failure	4	5.9
Distant lymph node metastases only	3	4.4
Distant organ metastasis only	8	11.8
Locoregional failure plus distant metastasis	2	2.9
Dead of complication	5	7.4
Other diseases	4	5.9
Unknown	3	4.4

### Toxicity

The incidence of grade 3 or higher acute radiation toxicity was seen in Table 3. Esophagitis in Grade 3 was recorded in 18 patients (26.4%). Leucopenia in grade 3–4 was recorded in 22 patients (32.4%). No patients developed grade 3 and worse lung toxicity (pneumonitis). Treatment-related high-grade (≥ 3) skin injury and gastrointestinal reaction were observed in 1 and 2 patients, respectively. One patient developed esophageal stenosis in grade 3, and another one had pulmonary fibrosis in grade 3. However, five patients died of late complications as displayed in Table 4, including gastrointestinal hemorrhage (4.4%) and esophagus fistula (2.9%).

**Table 3:** Treatment-related toxicity

**Treatment-related toxicity**	**Grade 0-1**	**Grade 2**	**Grade 3**	**Grade 4**	**Grade 5**
Acute toxicity					
Lung	67 (98.5%)	1 (1.5%)	0	0	0
Esophagus	29 (42.7%)	21 (30.9%)	18 (26.4%)	0	0
Skin	67 (98.5%)	0	1 (1.5%)	0	0
Hematologic					
Hemoglobin	60 (88.2%)	3 (4.4%)	4 (5.9%)	1 (1.5%)	0
Leucopenia	20 (29.4%)	26 (38.2%)	20 (29.4%)	2 (2.9%)	0
Neutropenia	53 (77.9%)	3 (4.4%)	12 (17.7%)	0	0
Thrombocytopenia	48 (70.6%)	13 (19.1%)	5 (7.4%)	2 (2.9%)	0
Gastrointestinal	57 (83.8%)	9 (13.2%)	2 (2.9%)	0	0
Late toxicity					
Lung	64 (94.1%)	3 (4.4%)	1 (1.5%)	0	0
Esophagus	61 (89.7%)	4 (5.9%)	1 (1.5%)	0	2 (2.9%)
Heart	66 (97.1%)	2 (2.9%)	0	0	0

**Table 4:** Treatment-related complications

**Complications**	**No**	**%**
Upper-gastrointestinal hemorrhage	3	4.4
Esophagus-tracheal fistula	2	2.9

## Discussion

ESCC remains one of the most lethal carcinomas in China. The poor survival is mainly related to the advanced stages at diagnosis. The optimal management for locally advanced disease remains controversial. Surgery or RT alone is associated with a poor survival, usually less than 10% at 5 years [16]. Randomized studies [1,2] have demonstrated the survival advantage of concurrent chemoradiotherapy compared with RT alone for patients with squamous cell or adenocarcinoma of the esophagus. Although concurrently administered chemotherapy acts as a promoter of the locoregional effects of RT, the locoregional control remains unsatisfactory in RTOG 85–01 trail [1]. Dose escalation trial by RTOG 94–05 failed to improve the locoregional control as well [2]. It may thus be concluded that by simply increasing the local radiation dose cannot easily convert to survival benefits for esophageal carcinoma patients. Therefore, many efforts have been made to explore the more reasonable treatment schedules based on this thinking and understanding to radiobiology.

From last two decades, a further increase in local-regional tumor control may be expected by augmenting radiation effects by altering fractionation schedule, because rapid tumor clonogen repopulation during treatment seems to be a cause of poor prognosis for cancer patients. One phase II study from Japan showed a promising result for ESCC patients treated with accelerated hyperfractionated RT plus 5-fluorouracil/cisplatin chemotherapy [17]. In China, Shi and their teams [18] initiated the study of LCAHRT for ESCC treatment, and yielded very encouraging results. Comparing with conventional fractionation (CF), the 5-year survival (34% versus 15%) and local control (55% versus 21%) were markedly improved with the LCAHRT regime. Henceforth, unremitting results from randomized and retrospective trials came out [19-23]. Recently, three independent meta-analysis from China strengthened the evidence of therapeutically beneficial of LCAHRT compared with CF for esophageal carcinoma [5,24,25].

Because of unique geographical and pathologic features of esophageal carcinomas in China, furthermore, esophageal carcinomas tend to spread axially to regional lymphatics, producing morbidity and mortality from locoregional effects [1]. Based on the previous studies [9,10], we supposed that elective HRLNR irradiation might be associated with lower nodal failure in advanced stage ESCC, and this hypothesis was supported by recent studies from Asian [11,12,26]. From another point of view, considering of high incidence of nodal failure in combined modality therapy, chemotherapy seems to play a limited role in preventing locoregional or distant nodal failure. Omitting ENI may be not reasonable for locally advanced stage ESCC patients, especially if the primary tumor located in lymphatic-rich regions [27] and PET/CT could not be incorporated in the planning of radiotherapy [28].

Previous study designed by Zhao et al. [22] enrolled 54 cases of local advanced stage ESCC with clinical stage T_1-4_N_0-1_M_0_ treated with LCAHRT (41.4Gy/23 fractions followed by 27Gy/18 fractions with 1.5Gy per fraction, twice a day) and 2 cycles of concurrent chemotherapy with cisplatin (25 mg/m^2^/day days 1–3) plus 5-FU (600 mg/m^2^/day days 1–3). And they reported the median survival was 30.8 months (95%CI, 17.6–44.1 months), Survival rates at 1, 3, and 5 years were 67%, 44%, and 40%, respectively. Twenty-six percent (14/54) of patients had locoregional disease presenting as the first failure, and distant metastases as the first failure occurred in 24% (13/54). In our study, 40Gy/20 fractions delivered to primary and metastatic regional tumor(s) and HRLNR, and additional 19.6Gy/14 fractions in 1.4Gy per fraction, twice a day, were given to the boost volume. Cisplatin-based chemotherapy concurrently administered during the RT course. Our regimen improved median survival time from 30.8 to 34.4 months, and the OS at 1-, 3-year improved from 67%, 44% to 75.5%, 46.5%, respectively. However, this trend was not observed at 5-year (40% vs. 22.7%). It should be noticed that approximately 32.3% of the population consisted of stage IV_a_ cases, and 46% of eligible patients were enrolled between 2010 and 2011 (Figure 1) in our study, which may partially explain the relatively poor 5-year OS. By subgroup analysis, for patients with stage II–III, the 5-year OS reached to 39.9%, which was similar to the results of Zhao et al. [22]. Despite the study cohort consisted of a high proportion of patients with stage IV_a_, this regimen was associated with only twenty-nine percent of local or/and regional failure and nineteen percent of distant metastases. Previous studies (Table 5) focusing on LCAHRT regimen reported the patterns of failure from local/locoregional failure and distant metastasis were 12.5–41.8% and 15.4–34.9%, respectively [18-23] in patients with TNM stage limited to I–III.

**Table 5:** Survival and toxicities of patients undergoing late course accelerated hyper-fractionated radiotherapy for esophageal squamous cell carcinoma

**First author**	**Study**	**No. of patients**	**TNM stage**	**Treatment scheme**	**Overall survival**	**Locol control**	**Treatment failure**	**Toxicity (Grade≥3)**
Shi XH [18]	Prospective	43	T_1-4_N_0-1_M_0_	41.4Gy/23fx followed by 27Gy/18fx with 1.5Gy, bid	5-year 34%	5-year 55%	LR 41.8% (18/43)	PI 27.9% (12/43)
							DM 18.6% (8/43)	EI 34.9% (15/43)
Wang Y [19]	Prospective	52	T_1-4_N_0-1_M_0_	41.4Gy/23fx followed by 27Gy/18fx with 1.5Gy,bid	1-year 80.0%	1-year 80.7%	LRF 13.5% (7/52)	PI 3.8% (2/52)
								EI 9.6% (5/52)
					3-year 41.2%	3-year 57.1%	DM 15.4% (8/52)	ES 1.9 % (1/52)
								PF 0% (0/48)
								EH 3.8% (2/52)
Zhao KL [20]	Retrospective	56	T_1-2_N_0_M_0_	41.4Gy/23fx followed by 27Gy/18fx with 1.5Gy, bid	1-year 90.9%	1-year 90.9%	LR 12.5% (7/56)	PI 5.4 % (3/56)
					3-year 54.6%	3-year 84.5%	DM 21.4% (12/56)	EI 10.7 % (6/56)
								ES 1.8 % (/56)
					5-year 47.8 %	5-year 84.5 %		PF 1.8 % (/56)
Zhao KL [21]	Retrospective	201	T_1-4_N_0-1_M_0_	41.4Gy/23fx followed by 27Gy/18fx with 1.5Gy, bid	1-year 73%,	1-year 77%	LRF 38.4% (77/201)	EI 15.4% (31/201)
								PI 7.0% (14/201)
					3-year 34%,	3-year 58%	DM 34.9% (70/201)	EF 0.5% (1/201)
					5-year 26%	5-year 56%		
Zhao KL [22]	Prospective	54	T_1-4_N_0-1_M_0_	41.4Gy/23fx followed by 27Gy/18fx with 1.5Gy, bid Plus ≥2 cycles of CHT with cisplatin+5-FU	1-year 67%,	1-year 84%	LRF 25.9% (14/54)	EI 24.1% (13/54)
								PI 5.5% (3/54)
					3-year 44%,	3-year 74%		PF 14.8% (8/54)
					5-year 40%	5-year 67%	DM 24% (13/54)	ES 3.7% (2/54)
Wang JH [23]	Prospective	48	T_1-4_N_0-1_M_0_	40Gy/20fx followed by 21–27 Gy/14-18fx with 1.5Gy, bid	1-year 79.2%	1-year 81.3%	LR 35.4% (17/48)	PI 33.33% (16/48)
								EI 27.1% (13/48)
					3-year 43.8%	3-year 50.0%	DM 16.7% (8/48)	ES 14.6% (7/48)
								PF 0% (0/48)

Note: distant metastasis–DM; local recurrence–LR; locoregional failure–LRF; esophagitis–EI; pneumonitis–PI; pulmonary fibrosis–PF; esophageal stenosis–ES; esophageal hemorrhage–EH; esophagus fistula–EF; fractions–fx; twice a day–bid; concurren chemotherapy–CHT.

Regarding tolerance in our study, high-grade (≥3) acute esophagitis and leucopenia were seen in 26.4% and 32.4% of patients, respectively. Similar findings were reported by Zhao et al. [22]. The higher rates of acute toxicities may be related to this treatment scheme. Our approach allowed us to avoid potential micrometastasis of lymphatics, nevertheless, it was gained at the expense of early toxicities. Therefore, how to minimize the damage to the sensitive normal tissues within irradiated fields without sparing cancer is a big challenge. Recent innovation of RT technology has made it possible to use sophisticated rotational intensity-modulated RT. This can deliver intensified radiation doses to the tumor while minimizing the doses to the normal tissues [29]. Acceleration of radiation treatment involves the delivery of the target dose in less time, and is analogous to the concept of dose-intensity in the delivery of cytotoxic chemotherapy. In order to prevent late toxicity, we reduced the dosage of per fraction (1.4Gy per fraction twice day). The interval between daily fractions (typically ≥ 6 hours) provides normal tissues with time to repair sublethal radiation damage, which may be responsible for the lower rate of late lung toxicity in our study. Notably, treatment-related upper-gastrointestinal hemorrhage (4.4%) and esophagus-tracheal fistula (2.9%) were relatively higher, which should be paid much attention. Although the reason for the higher incidence of such complications was unknown, we suspected that it might be association with advanced T stage in our cohort of patients.

Several strengths and limitations should be noted. This was a single center study with a relatively small sample size, which may limit the generalizability of our findings. This study cohort consisted of a higher proportion of stage IV_a_ who had worse prognosis. Moreover, a variety of cisplatin-based chemotherapy regimens were enrolled, although OS rate was not associated with combined regimens. The chemotherapy agents are usually 5-fluorouracil and cisplatin. Recently, capecitabine and pemetrexed have been introduced in phase I and II trials and the preliminary results are promising [13]. Therefore, how to best combine chemotherapy and accelerated radiotherapy regimens to maximize local control and survival is an important question. Further trials and observation confirming the efficiency of LCAHRT concurrently with these chemotherapy regimens are still needed. At last, this accelerated radiation scheme is broadly used as a standard treatment for locally advanced ESCC and non-small cell lung cancer in China, however, has not met with widespread clinical acceptance in the United States or Europe probably because of higher acute toxicity, and the use of twice fractions per day.

In conclusion, this phase II study with limited number of patients demonstrated that ENI LCAHRT concurrently with CHT was generally tolerated, and the treatment outcome was satisfactory. Although these results by this interim analysis were not sufficient to confirm the impact on ESCC treatment with this regimen, the results in our cohort patients with a higher proportion of advanced stage were compared favorably with those of previous studies. Further observation with longer time follow up and randomized phase III trial is currently underway.

## Abbreviations

LCAHRT: Late course accelerated hyper-fractionated radiotherapy; ESCC: Esophageal squamous cell carcinoma; ENI: Elective lymph node irradiation.

## Competing interests

The authors declare that they have no competing interest.

## Authors' contributions

DW, JY and MS carried out the manuscript writing; JZ participated in statistical analysis; BL and LZ conceived and designed this study; HG, TZ, YW, WH, ZW, HL, and ZZ helped to collect data. All authors read and approved the final manuscript.
